# Childhood overweight, obesity and associated factors among primary school children in dire dawa, eastern Ethiopia; a cross-sectional study

**DOI:** 10.1186/s40608-017-0156-2

**Published:** 2017-06-01

**Authors:** Assefa Desalew, Alemnesh Mandesh, Agumasie Semahegn

**Affiliations:** 1 School of Nursing and Midwifery, College of Health and Medical Sciences, Haramaya University, Po. Box. 235, Harar, Ethiopia; 2 Department of Nursing and Midwifery, School of Allied Health Sciences, College of Health Sciences, Addis Ababa University, Addis Ababa, Ethiopia

**Keywords:** Overweight, Obesity, Children, School, Dire Dawa, Ethiopia

## Abstract

**Background:**

Obesity in children is increasing worldwide. Malnutrition has become a double burden challenge of public health concern in developing countries. Childhood obesity increases the risk of chronic disease in childhood as well as adulthood. However, information is very scarce about childhood obesity in developing countries specifically in Ethiopia. Therefore, we aimed to assess childhood overweight, obesity and associated factors among primary school children at Dire Dawa, Eastern Ethiopia.

**Methods:**

A school based cross-sectional study was conducted in Dire Dawa from 1^st^ to 30^th^ March, 2016. Study participants were selected using multistage sampling method. Pre-tested self-administered questionnaire, face to face interview technique and anthropometric measurements were used to collect data by eight well trained data collectors. Data were coded, cleaned and entered into EpiData software version 3.5.1, and exported into SPSS (version 21.0) statistical software, for data analysis. Bivariate and multivariate logistic regression were carried out to identify associated factors with childhood overweight and obesity. Statistical significance was declared using Adjusted Odds Ratio (AOR) at 95% CI and *p*-value less than 0.05.

**Results:**

The prevalence of overweight and obesity were 14.7% (95% CI: 11.7, 18.0) and 5.8% (95% CI: 3.6, 8.0), respectively. Children who were from private school (AOR = 3.4, 95% CI: 1.4, 8.5), from families belonged to high socioeconomic class (AOR = 16.9, 95% CI: 6.5, 23.9), preferred sweetened foods (AOR = 2.3, 95% CI: 1.1, 5.1), had not engaged in regular physical exercise (AOR = 3.8, 95% CI: 1.5, 9.8), had experienced sedentary life style like spent their free time watching TV (AOR = 3.6, 95% CI: 1.6, 7.9), play computer game (AOR = 4.6, 95% CI:1.4,15.4), and were not having close friends (AOR = 2.9, 95% CI: 1.4, 6.2) were significantly associated with overweight/obesity risk.

**Conclusion:**

Overweight**/**obesity in children is on alarming stage in the study area. Therefore, more attention should be given to creating awareness about healthy diet and improving life style through school and public media in collaboration with concerned bodies.

## Background

A combination of obesity and overweight represents a severe public health problem. Globally, the trend of obesity prevalence and its impact has increased in the society, especially in the recent times [1, 2]. It has more than doubled worldwide since 1980. Globalization, improving economic conditions and changing dietary habits in developing countries are responsible risk factor for the rapid increase in obesity. Currently, the estimates shows that overweight in children is increasing in developing countries, and obviously high in developed countries [1, 3]. In the meantime, the intake of energy-dense foods which contain high fat, salt and sugars have been increasing. While, low intake of micronutrients and having sedentary life style have become a risk for overweight and obesity in connection with increase in urbanization [4–6].

On the other hand, school based health promotion approaches such as improving children’s physical activity, fruits and vegetables intake, have been found helpful to minimize risk of childhood obesity [7]. Studies have reported that about 50–80% of children who develop obesity early in life, end up becoming obese in later life [8]. Globally, obesity in childhood have been described as double burden of malnutrition which has multiple consequences, and it is known to be associated with adulthood chronic illness. It leads to morbidity such as risk of high blood pressure, diabetes mellitus, respiratory disease, dyslipidemia, tumor and orthopedic disorders [9–19].

Childhood under nutrition and obesity are simultaneous occurrence in many developing countries [19–21]. In many Sub-Saharan Africa countries, research and investment in health has been mainly focused on infectious diseases and under nutrition. In the meantime, obesity is a well-recognized risk factor for various chronic health problems, and also predictor of future adulthood health status [22–24]. The factors associated with overweight and obesity are mainly entrenched with health behaviors, dietary habits, daily physical activity, broader social, environmental and biological determinants [25, 26].

Though there are few studies conducted in Ethiopia, they revealed that childhood overweight and obesity are emerging and consistently increasing in magnitude particularly in private school children [27–29]. The fact that there is a growing demand to prevent non-communicable diseases starting at early ages. Nevertheless, information is very scarce about childhood obesity in developing countries specifically in Ethiopia. Most of the research focused on undernutrition assessment. We could not find a study done in Dire Dawa city during our literature review from September 2015 to March 2016. Therefore, this study was designed to determine the magnitude and associated factors of childhood overweight and obesity. The study will elevate the need for program planners, policy makers, parents or guardians, clinicians and all other stakeholders to give an emphasis for childhood overweight and obesity. Therefore, we aimed to assess childhood overweight, obesity and associated factors among primary school children at Dire Dawa, Eastern Ethiopia.

## Methods

### Study setting and study design

A school based cross-sectional study design was employed using quantitative method to assess the level of childhood overweight and obesity. The study was conducted in Dire Dawa city administration, from 1^st^ to 30^th^ March, 2016. Dire Dawa is one of the two Federal Administrative Cities in the Federal Democratic Republic of Ethiopia. Dire Dawa city is located about 515 km Eastern direction from Addis Ababa. At the time of the interview, a total of 38,376 school age children were living in the city. In Dire Dawa city, in-school children from 63 primary schools that had 5th to 8th grades were eligible for this study. Children who had any chronic disease, mental illness and physical disability were not included into the study.

### Sample size and sampling procedure

Sample size was calculated using single population proportion formula considering proportion of overweight of ten percent in Addis Ababa primary school children [29], 95% Confidence Interval (CI) and five percent margin of error. Ten percent for non-response rate and a design effect of three were considered to obtain representative sample size. Finally, a total of 456 participants were obtained. Multistage sampling methods were used to select the study participants. Henceforward, primary schools were stratified into public and private schools in which we assumed obesity could vary across public and private schools. A total of eight primary schools; five from public and three from private were selected randomly from eligible primary schools. A total of 6,587 children were enumerated from the recruited schools. The sample size was proportionally allocated to size into public and private schools accordingly. Sampling frame was prepared based on students class room register. Then participants were selected using systematic sampling method with every ‘K^th^’ interval (k = 14). Finally, 456 school going child-parent pairs were included into the study.

### Data collection tools and quality control

Data were collected using both structured and semi-structured questionnaires. The questionnaire was comprised of socio-demographic characteristics, physical activity, sedentary life style factors, and dietary habits related questions. In addition, anthropometric measures (weight and height) were carried out. Most of the questionnaires were adapted, and modified to suite and relate to the study objective and area’s context. Weight was measured by using UNICEF seca digital balance scale and recorded to nearest 0.5 Kg. Children’s height was measured using portal stadiometer. Enumerators strictly followed appropriate height measuring procedures. Height was measured by placing a child in standing position that heels, buttocks, scapula/upper body parts and occiput touching the measuring board. Height measurement values were read and recorded to nearest 0.1 cm. The weight and height of the children were measured with barefoot. The children’s weight was measured using weight measurement balance in wearing only light clothing and barefoot. Children’s bags, books, exercise books and other playing materials were put away by data collectors. Body Mass index (BMI) was calculated using Microsoft Excel 2013, and applying a mathematical formula where weight in kilograms (kg) divided by height in meter square (m^2^). BMI-for-age percentiles for age were computed separately for male and female using Center for Disease Control (CDC) growth charts developed in 2000 [30]. To assure the quality of data, the data collectors and supervisors were trained for one day. The training addressed the sampling procedures, data collection methods, consent and assent taking, understanding the questionnaire, and purpose of the study. Meanwhile, a pretest of the instrument was carried out in one of the nearby primary school in Harar city. The anthropometric measurement tool calibrations and close supervision of data collection process were carried out. One day orientation was given to eight school teachers about the data collection procedures and instruments. The questionnaire was developed in English, and then translated into the local language (Amharic). The language review was made by experts for consistency of translation.

### Data processing and analysis methods

Data were coded, manually checked and entered into EpiData software version 3.5.1, and exported into SPSS (version 21.0) statistical software. Data were cleaned by running frequency to explore the variables one by one. Data anomalies, typos and other errors were corrected by cross-checking against the originally filled hard copy questionnaire. Data were analyzed with SPSS (version 21.0) statistical software. Descriptive statistics was computed to determine the magnitude of the explanatory variables and outcome variable. Binary logistic regression was done to investigate the association between explanatory variables and outcome variable (childhood overweight and obesity). Explanatory variables such as maternal education, occupation, religion, average family income, family size, family car ownership, school type, monthly income, vigorous-sports, spend free time, sleeping habits in the afternoon, having close friends, food preferences (such as sweetened foods, fruits, vegetables, snacks, number of meals per day) were included into the binary regression. Of these, explanatory variables which had crude odds ratio at 95% CI and *p*-value of less than 0.2 on the bivariate analysis were included into the final multiple logistic regression model to control for confounders. Multiple logistic regression was tested for model fitness by using Hosmer-Lemshow model test. Multicollinearity of the independent variables was checked by standard error, and variables with standard error of greater than two were excluded from the multivariate analysis. Finally, adjusted odds ratio with 95% CI at *p*-value less than 0.05 was used to declare statistically significant association.

## Results

### Parental socio-demographic characteristics

A total of 448 children-parent pairs were assessed in this study. The response rate was 98.2%. The mean family monthly income was 3,014 (±1,307.9) Ethiopian Birr. Three hundred twenty (71.4%) of parents were female, and 422 (41.1%) of them attended primary education. One hundred seventy six (39.3%) of mothers were house wives by occupational status. One third (34.8%) of them had involved in private business for the last 12 months preceding this study. Almost one out of ten children’s family had their own car, which they used for transportation. Approximately three quarter (72.8%) of the parents had five and less family size (Table 1). Regarding to maternal history of gestational diabetes mellitus and family history of chronic disease, 11 (2.5%) of mothers had history of gestational diabetes mellitus, and 48 (10.7%) of parents had history of chronic diseases.

**Table 1:** Sociodemographic characteristics of parents among primary school children in Dire Dawa City, Eastern Ethiopia, March 2016 [*n* = 448]

Variables		Frequency	Percent
Respondent’s sex	Male	128	28.6
	Female	320	71.4
Maternal educational status	No formal education	49	10.9
	Primary level	184	41.1
	Secondary level	145	32.4
	College or University	70	15.6
Maternal occupation	House wife	176	39.3
	Government employee	116	25.9
	Private business	156	34.8
Religion	Orthodox	173	38.6
	Muslim	174	38.8
	Catholic	40	8.9
	Protestant	55	12.3
	Others	6	1.3
Average family monthly income	Below the mean	240	53.6
	Above the mean	208	46.4
Family size	Less than or equal to 5	326	72.8
	Greater than 5	122	27.2
Family own car	Yes	41	9.2
	No	407	90.8

### Children’s sociodemographic characteristics

Among a total of 448 children, 187 (41.7%) were male and 261 (58.3%) were female. The mean age of the children was 13.1 (±1.4) years. Regarding to children’s grade level, 115 (25.7%), 138 (30.8%), 121 (27.0%) and 74 (16.5%) of the children were 8th, 7th, 6th and 5th grades, respectively. Almost three quarter (72.5%) of children were from government schools.

### Magnitudes of overweight and obesity

**Fig. 1:**
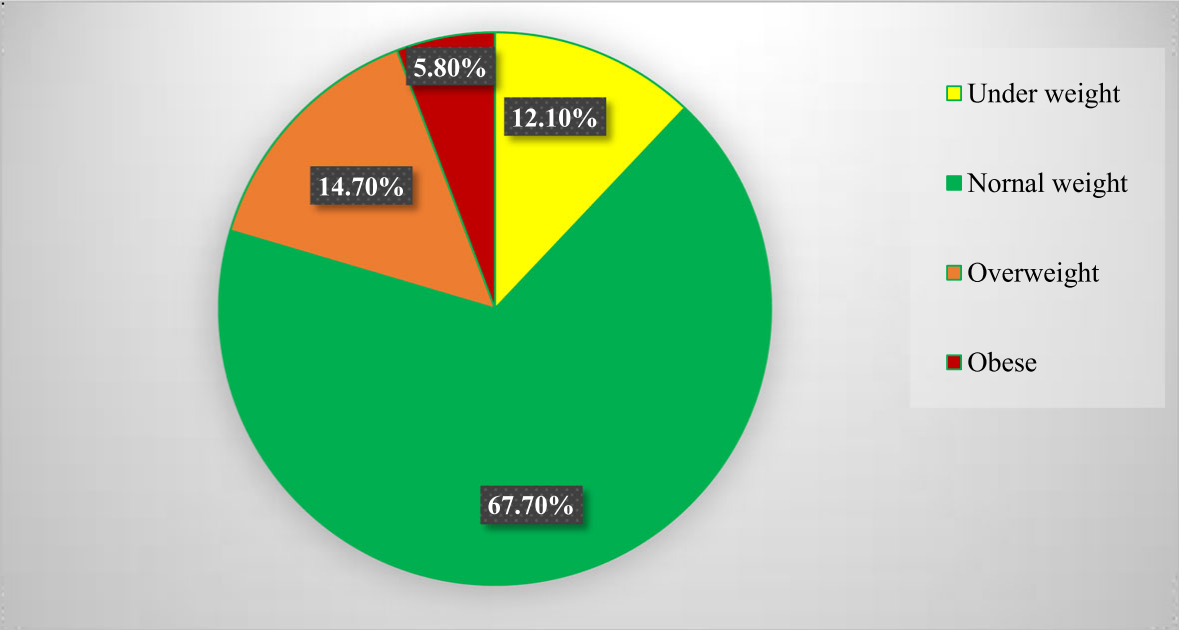
BMI of primary school children in Dire Dawa City, Eastern Ethiopia, March, 2016 [n = 448]

The overall magnitude of underweight, normal weight, overweight and obesity were 12.1, 67.4, 14.7 and 5.8%, respectively (Fig. 1). The prevalence of overweight and obesity were 14.7 and 5.8%, respectively. Meanwhile, females (16.5%) were more overweight than male children (12.3%). Nevertheless, males (8.6%) were more obese than female children (3.8%). Children attended at government (9.2%) and private schools (29.3%) were overweight. Similarly, 1.8% of government and 16.3% of private schools children had childhood obesity (Table 2).

**Table 2:** Sociodemographic characteristic of children according to Body Mass Index among primary school children in Dire Dawa City, Eastern Ethiopia March, 2016 [*n* = 448]

Variables		Child body mass index			
		Underweight	Normal weight	Overweight	Obese
Sex	Male	22 (11.8%)	126 (67.4%)	23 (12.3%)	16 (8.6%)
	Female	32 (12.3%)	176 (67.4%)	43 (16.5%)	10 (3.8%)
Age	11 years	12 (18.8%)	43 (67.2%)	9 (14.1%)	0 (0.0%)
	12 years	14 (12.7%)	75 (68.2%)	17 (15.5%)	4 (3.6%)
	13 years	9 (10.6%)	63 (74.1%)	10 (11.8%)	3 (3.5%)
	14 years	12 (13.0%)	57 (62.0%)	13 (14.1%)	10 (10.9%)
	15 years	7 (7.2%)	64 (66.0%)	17 (17.5%)	9 (9.3%)
Grade	Grade five	12 (16.2%)	51 (68.9%)	10 (13.5%)	1 (1.4%)
	Grade six	14 (11.6%)	83 (68.6%)	19 (15.7%)	5 (4.1%)
	Grade seven	17 (12.3%)	90 (65.2%)	20 (14.5%)	11 (8.0%)
	Grade eight	11 (9.6%)	78 (67.8%)	17 (14.8%)	9 (7.8%)
Family own car	Yes	0 (0.0%)	13 (31.7%	18 (43.9%)	10 (24.4%)
	No	54 (13.3%)	289 (71.0%)	48 (11.8%)	16 (3.9%)
School ownership	Government	47 (14.5%)	242 (74.5%)	30 (9.2%)	6 (1.8%)
	Private	7 (5.7%)	60 (48.8%)	36 (29.3%)	20 (16.3%)

### Dietary habits and food preference

**Fig. 2:**
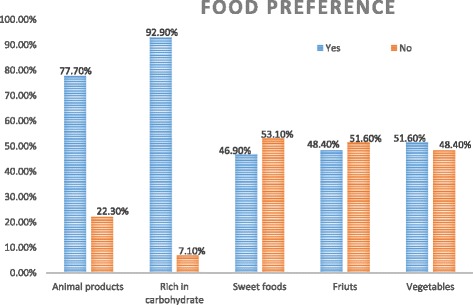
Distribution of food preferences of the primary school children in Dire Dawa City, Eastern Ethiopia, March, 2016 [*n* = 448]

Regarding the dietary habits of children, 176 (39.3%) and 170 (37.9%) of them had not consumed fruits and vegetables, respectively. Of these, majority (96.0%) of them reported having food more than once per day. While more than half (54.9%) of them usually had snack. Children had a habit of buying junk food. Cake, biscuit, ice cream and chocolate were some of the common food items that were bought by children 28.1, 47.1, 17.9, and 22.3%, respectively. Approximately half of the children (49.6%) used the most favorite local foods called “Baklawa”, “Wushebek” or “Halewa”. *Note that “Baklawa” or “Wushebek” or “Halewa” are local food items that made up of groundnut, plenty sugar, oil, refined wheat powder and other sweetening agents*. Forty eight (10.7%) and 126 (28.1%) of them ate their food while they were watching movies and or TV programs, respectively (Table 3). Nine out of ten children (92.9%) preferred much sweetened food items which are rich in carbohydrate (sugar) (Fig. 2).

**Table 3:** Eating habits and preferred foods among primary school children in Dire Dawa City, Eastern Ethiopia, March, 2016 [*n* = 448]

Variable		Frequency	Percent
Fruits consumption per week	Did not consume	176	39.3
	1-2 days per week	203	45.3
	3 and more days per week	69	15.4
Vegetables consumption per week	Did not consume	170	37.9
	1-2 days per week	215	48.0
	3 and more days per week	63	14.1
Where are you getting lunch?	Home	383	85.5
	School cafeteria	30	6.7
	Nearby food service establishment	35	7.8
Foods buying habits when going to movie or cinema	Yes	48	10.7
	No	136	30.4
	Did not go to movie	264	58.9
Do you eat while you Watching TV?	Yes	126	28.1
	No	308	68.8
	Did not watch TV	14	3.1
Do you eat while studying?	Yes	48	10.7
	No	400	89.3

### Physical activity and sedentary lifestyle

**Fig. 3:**
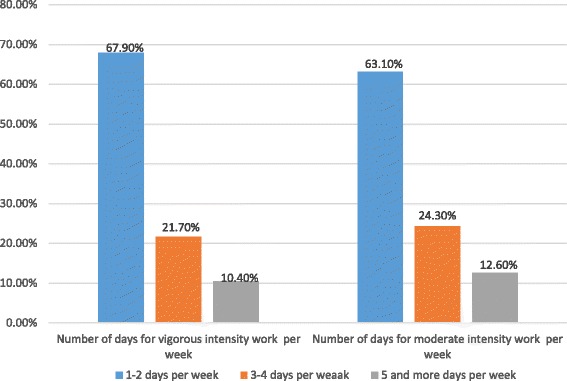
Distribution of number of day for vigorous and moderate intensity exercises among primary school children in Dire Dawa City, Eastern Ethiopia, March 2016 [*n* = 448]

Only a quarter (25.2%) of children did some physical activities besides learning. Of these, and children engaged in vigorous 106 (93.8%), and moderate intensity 111 (98.2%) exercises for at least 10 min every day. Children spent only a few minutes for physical activities. Sixty four (34.9%), 30 (32.6%) and 12 (32.6%) of them spent their time on vigorous exercises for less than 60, 60-120 and greater than 120 min, respectively (Fig. 3).

More than half (58.3%) of children had habits of walking on foot and also ride bicycle. Of these, more than one third (34.9%) of them had walking or riding bicycle for 1-2 days per week. Eighty three (50.9%) of the children exercised vigorous sport for 1–2 days per week, while 43.6% of them spent 60–120 min exercising. On the other hand, 55.4, 31.0 and 8.9% of children spent their free time in resting or just reading books, watching Television (TV), different videos and playing computer games, respectively (Table 4). In additional, majority of them 396 (88.4%) had no habits of taking short nap in the afternoon. While for average regular sleep duration, 5.4 72.3 and 22.3% of them slept for less than 7 hours, 7 to 8 hours and greater than or equal to nine hours per day, respectively. Only 10.3% of the children felt unhappy most of the time. Nevertheless, around half of the participants (50.9%) had close friends in school or in their neighborhoods.

**Table 4:** Physical activity and sedentary life style among primary school children in Dire Dawa City, Eastern Ethiopia, March, 2016 [*n* = 448]

Variables		Frequency	Percent
Have you ever had walk on foot or ride bicycle for at least 10 min	Yes	261	58.3
	No	187	41.7
How many days do you walk or use a bicycle per week	1-2 days	91	34.9
	3-4 days	85	32.6
	5 and more days	85	32.6
Time spend in walking or bicycling per day	Less than 30 min	104	39.8
	30-59 min	91	34.9
	60-90 min	55	21.1
	Greater than 90 min	11	4.2
Vigorous-intensity sports for at least 10 min	Yes	163	36.4
	No	285	63.6
Number of days for vigorous activities/sports per week	1-2 days per week	83	50.9
	3-4 days per week	58	35.6
	5 and more days per week	22	13.5
Time spend doing vigorous physical activities/sports per day	Less than 60 min	68	41.7
	60-120 min	71	43.6
	Greater than 120 min	24	14.7
Have you ever had moderate-intensity sports for at least 10 min	Yes	158	96.9
	No	5	3.1
Number of days for moderate physical activities/sports per week	1-2 days per week	71	44.9
	3-4 days per week	62	39.2
	5 and more days per week	25	15.8
Time spend for moderate physical activities/sports per day	Less than 60 min	64	40.5
	60-120 min	70	44.3
	Greater than 120 min	24	15.2
How do you spend your free time?	Reading books	248	55.4
	Watching TV, video	139	31.0
	Playing computer game	40	8.9
	Others	21	4.7
How long do you spend sitting per day?	Less than three hours	172	38.4
	3-5 h	237	52.9
	6 and more hours	39	8.7

### Factors associated with childhood overweight and obesity

A total of thirteen explanatory variables were assessed, six of them were found to be significantly influence child overweight and obesity. Children who learn in private schools were almost three times more likely to be overweight or obese as compared to those learn in government schools (AOR = 3.4, 95% CI:1.4, 8.5). Children who preferred sweetened foods were almost two times more likely to be overweight or obese as compared to those who did not preferred sweetened foods (AOR = 2.3, 95% CI:1.04, 5.1). Children who did not perform vigorous intensity sports were about four times more likely to be overweight and obese as compared to those who did vigorous intensity sports for at least 10 min per activities (AOR = 3.8, 95% CI:1.5, 9.8). Regarding to children who spent their free time; by watching TV (AOR = 3.6, 95% CI:1.6, 7.9), and playing computer (AOR = 4.6, 95% CI:1.4, 15.4) were almost four times more likely to be overweight and obese compared to those who did not spend time in watching TV and playing computer games (Table 5).

**Table 5:** Association between explanatory variables and childhood overweight and obesity among primary school children in Dire Dawa City of Eastern Ethiopia, March 2016 [*n* = 448]

Variables		Frequency	Overweight and Obesity		COR (95% CI)	AOR (95% CI)	*P* value
			Yes	No			
Maternal occupation	Housewife	176	29	147	1.0	1.0	
	Employee	116	22	94	1.2 (0.6, 2.2)	1.2 (0.5, 3.2)	0.727
	Private business	156	41	115	1.8 (1.1,3.1)	1.2 (0.5, 2.6)	0.881
Family own car	Yes	41	28	13	11.5 (5.7,23.5)	1.1 (0.4,3.4)	0.864
	No	407	64	343	1.0	1.0	
School type	Government	325	36	289	1.0	1.0	
	Private	123	56	67	6.7 (4.1,11.1)	**3.4 (1.4,8.5)**	**0.007**
Monthly income	Below the mean	240	6	234	1.0	1.0	
	Above the mean	208	86	122	17.5 (11.7,34.7)	**16.9 (6.5,23.9**)	**0.000**
Vigorous-sports	Yes	163	9	154	1.0	1.0	
	No	285	83	202	7.0 (3.4,14.4)	**3.8 (1.5, 9.8)**	**0.005**
Spend your free time	Reading	269	16	253	1.0	1.0	
	Watch TV, video	139	49	90	8.6 (4.7,15.9)	**3.57 (1.6,7.9)**	**0.002**
	Computer Play	40	27	13	22.8 (14.3,35.5)	4.6 (1.4,15.4)	**0.013**
Sleeping in afternoon	Yes	52	29	23	6.7 (3.6,12.3)	2.6 (1.0,6.7)	**0.049**
	No	396	63	333	1.0	1.0	
Having close friends	Yes	228	33	195	1.0	1.0	
	No	220	59	161	2.2 (1.4,3.5)	**2.9 (1.4,6.2**)	**0.004**
Sweetened foods	Yes	210	61	149	2.7 (1.7, 4.4)	**2.3 (1.1, 5.1)**	**0.039**
	No	238	31	207	1.0	1.0	
Fruits	Yes	217	38	179	1.0	1.0	
	No	231	54	177	1.4 (0.9, 2.3)	1.2 (0.3,5.4)	0.788
Vegetables	Yes	231	41	190	1.0	1.0	
	No	217	51	166	1.4 (1.0, 2.3)	1.3 (0.3, 5.1)	0.742
Snack	Yes	246	58	188	2.1 (1.1,4.2)	1.6 (0.7,3.3)	0.237
	No	202	34	168	1.0	1.00	
Number of meals per day	<3 times	28	9	19	1.00	1.00	
	> = 3 times	420	83	337	1.9 (0.8, 4.4)	0.8 (0.2,2.9)	0.712

Explanatory variables such as maternal education, religion and family size were excluded after binary regression

## Discussion

The findings of the present study determined the magnitude and associated factors of overweight and obesity among primary school children in Dire Dawa city administration, eastern Ethiopia. Accordingly, the overall prevalence of underweight, normal weight, overweight and obesity were 12.1, 67.4, 14.7 and 5.8%, respectively. The findings of the present study were almost consistent with another study conducted in Ethiopia (2014) which revealed that underweight, normal weight, overweight and obesity are 9.5, 77.8 9.9 and 2.8%, respectively [29]. The frequency of overweight was higher in female (16.5%) than male (12.3%) children, but males (8.6%) were more obese than females (3.8%). The present study findings support findings reported in India which was conducted from 2007 to 2010 [31], and in Ethiopia [4]. Congruence in findings could be explained by similarity in the culture of Indian and Ethiopian societies in which females spent most of their time at home and more boys’ participate in field activities.

The finding of the present study was lower in magnitude of overweight and obesity compared to a study done in Australia [32]. Moreover, the finding of this study was also lower than studies done from 2008-2010 in United Arab Emirates and Bangladesh [20, 33] and in other African countries like in Egypt, South Africa and Kenya, but nearly similar with report from Cameron [3, 5, 34, 35]. This might be explained by the difference in feeding habits and socio-economic status and difference in standard for the cutoff point and sample size of participants. Regarding to school type; government and private schools, the level of childhood overweight and obesity in the present findings were consistent with studies in Kenya and Ethiopia (2014) [4, 23]. Majority of children preferred animal product, food rich in carbohydrate and sweets foods. However, the present findings are more comparable with studies done in Pernambuco (2011), Brazil (2015) and Kenya (2012) [35–37]. Conversely, the study done in Ethiopia (2015) revealed that (90%) preferred animal products, (88.6%) preferred rich foods in carbohydrate, and (17.7%) preferred sweetened foods, which was similar to the findings reported in the current study, even though higher preference of sweetened food were found in this study. This higher sweetened food preference might be due to culture of eating locally available sweetened foods in the community.

According to this study, children from private school were three times more likely to be overweight or obese compared to government schools children. The present study finding is consistent with studies in different countries from 2011 to 2015, in Brazil [37], India [38], Kenya [23], Ghana [16], and Ethiopia and [4, 28, 29]. This study also found that children from parental average monthly income above the mean were almost 17 times more likely to be overweight or obese compared to those family who had average monthly income below the mean score. The present study finding is consistent with studies done in China [39], Bangladesh [15], Romania [40], Brazil [15] and Egypt 2013 [5]. Similarly, the study findings also revealed, children, learning in private schools and belonging to parents with high socioeconomic status were strongly associated with overweight and obesity. One possible explanation might be, their higher socioeconomic status of private school children would allow them for higher adoption of unhealthy nutritional habits (fast foods, energy-dense snacks, sweetened foods, more animal products, etc) than other school children.

Likewise children who preferred sweetened foods were more likely to be overweight or obese compared to those who did not prefer sweetened foods. This was similar with different studies done in Europe as reported from a study conducted by World Health Organization (WHO) [41], India [42], Egypt [5], Tamale metropolis of Ghana [16], Kenya [35], and Ethiopia [28, 29] which revealed that sweetened foods preference were found significantly associating with overweight and obesity. This could be explained as sweetened food items are calorie dense foods which result in positive energy balance to their consumers. As per the findings from this study, children who had sleeping habit in the afternoon were 2.5 times more likely overweight and obese than children who did not sleep in the afternoon. Children who had no close friends in school or around their neighborhoods were almost three times more likely to be overweight and obese compared to those who had close friends. This was consistent with studies done in China [39], Ghana and Uganda [22]. In contrary to the findings of this study, a study conducted in China [43] revealed that short sleep duration and sleeping in the afternoon was not associated with obesity. This discrepancy might be due to difference in sleeping habits and age of the participants.

This study revealed that children who spent their free time by watching TV and playing computer were about four times more likely to be overweight and obese compared to those who spent their time reading. This finding confirms the findings from studies conducted in Brazil [44], Germany [45], Ghana [16], and Ghana and Uganda [22] that revealed children who spent their free time in viewing TV, play computer game for three and more hours were more likely to be overweight and obese. This might be explained by advancement in technology which has tremendously changed the life style of children. Watching TV and playing computer game may decrease the amount of time spent on playing outdoor games which might resulted in weight gain.

Likewise this study, revealed that children who did not perform vigorous intensity sports were about four times more likely to be overweight and obese compared to those who did vigorous intensity sports for at least ten minutes. This was similar with study done in Brazil [44], Ghana [16], Ghana and Uganda [22]. Several other studies conducted in Ghana [46], Egypt [5] and Ethiopia [4] found that vigorous intensity sports reduced risk of overweight and obesity. But other studies in Lithuania [47] and Romania [40] reported that any physical activities were not associate with reduction of the risk of increased overweight. This could be explained as physical activity results in energy expenditure thereby decreasing adiposity in the body.

### Strength and limitation of the study

This study used a probability sampling procedure to select study participants. We used questionnaire method and anthropometric measurement (height and weight) to assess the level of obesity. Since the study was cross-sectional, it was not possible to strongly to demonstrate cause and effect relationship. It used self-reporting (interview response) which might have social desirability bias. Some questions also required the participants to recall, which could have affected the results as most of them, could have forgotten.

## Conclusion

Both under nutrition and over nutrition coexist among primary schools children in Dire Dawa city. This study revealed that one in seven children were overweight. Approximately one in twenty children had obesity. The prevalence of overweight and obesity problem is slightly higher among girls than boys. Learning in private school, high parental socioeconomic class, sweet foods preference, physical inactivity or not engaging in sport exercise, sedentary life style like spent free time in viewing TV and play computer game, sleeping habit in afternoon and not having close friends were significantly associated with childhood overweight and obesity. We suggest integrated nutrition program should be implemented at school, and in the community with existing community based health extension program. Nutritional behavioral change communication should be delivered to parents and in school for children.

## Abbreviations

AOR: Adjusted Odds Ratio
BMI: Body Mass Index
CDC: Center for Disease Control
CI: Confidence Interval
COR: Crude Odds Ratio
SPSS: Statistical Packages for Social Sciences
TV: Television
UNICEF: United Nations Children’s Fund
WHO: World Health Organization
